# The stem region of group A transferase is crucial for its specificity, and its alteration promotes heterologous Forssman synthase activity

**DOI:** 10.1038/s41598-023-40900-4

**Published:** 2023-08-26

**Authors:** Emili Cid, Miyako Yamamoto, Laura Barrero, Fumiichiro Yamamoto

**Affiliations:** Laboratory of Immunohematology and Glycobiology, Josep Carreras Leukaemia Research Institute, Ctra. de Can Ruti, Cami de Les Escoles S/N, 08916 Badalona, Spain

**Keywords:** Biochemistry, Cell biology, Molecular biology

## Abstract

Some stem region mutants of human blood group A transferase (hAT) possess Forssman synthase (FS) activity, but very little is known about the mechanisms responsible for this enzymatic crosstalk. We performed confocal microscopy and image analysis to determine whether different intra-Golgi localization was accountable for this acquired activity. We also performed structural modeling and mutational and normal mode analyses. We introduced new mutations in the stem region and tested its FS and AT activities. No differences in subcellular localization were found between hAT and FS-positive mutants. AlphaFold models of hAT and mFS (mouse Forssman synthase) showed that the hAT stem region has a tether-like stem region, while in mFS, it encircles its catalytic domain. In silico analysis of FS-positive mutants indicated that stem region mutations induced structural changes, decreasing interatomic interactions and mobility of hAT that correlated with FS activity. Several additional mutations introduced in that region also bestowed FS activity without altering the AT activity: hAT 37–55 aa substitution by mFS 34–52, 37–55 aa deletion, and missense mutations: S46P, Q278Y, and Q286M. Stem region structure, mobility, and interactions are crucial for hAT specificity. Moreover, stem region mutations can lead to heterologous Forssman activity without changes in the catalytic machinery.

## Introduction

The human blood group ABO system (ABO) is composed of A and B antigens expressed on erythrocytes and antibodies against these antigens present in the sera of individuals who do not express the antigen(s) (Landsteiner’s Law)^1, 2^. A and B functional alleles at the ABO genetic locus encode α-1,3-*N*-acetylgalactosaminyltransferase (blood group A transferase, AT) and α-1,3-galactosyltransferase (blood group B transferase, BT), which catalyze the last steps of A and B antigen biosynthesis using H substance as common precursor substrate. AT and BT differ in 4 amino acids (aa), and aa 266 (Leu in AT, Met in BT) and 268 (Gly in AT and Ala in BT) are crucial for sugar specificity and enzymatic activity^3^.

**Figure 1:**
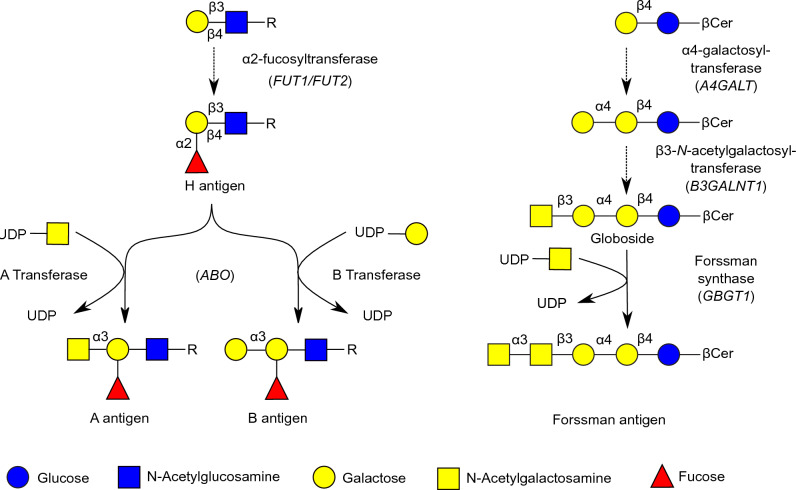
Schematic representation of biosynthetic pathways of blood group A, B, and FORS1 antigens. The biosynthetic pathways of blood group A, B, and FORS1 antigens are schematically shown. The monosaccharides are represented using the SNFG symbol nomenclature, and the glycosidic linkages are abbreviated (α2 for α1- > 2). Gene symbols are shown in between parentheses.

Forssman system (FORS) is a blood group system consisting of glycolipid Forssman antigen (FORS1) and antibodies against it^4^. Humans are a FORS1-negative species^5^; that is, most individuals do not express FORS1 antigen^4, 6^. However, there are FORS1-positive (FORS1 +) individuals who carry the activating mutation c.887G > A (rs375748588), although with an extremely low allele frequency (0.00001772)^7^. After this discovery, the International Society of Blood Transfusion (ISBT) accepted the FORS system as a *bona fide* blood group. Globoside α-1,3-*N*-acetylgalactosaminyltransferase 1, also known as Forssman glycolipid synthase (FS), is encoded by the functional GBGT1 allele^6, 8^ and catalyzes the last step in the FORS1 biosynthesis^9^. Biosynthetic pathways of blood group A, B, and FORS1 antigens are schematically shown in Fig. 1.

Mouse FS (mFS) GlyGlyAla tripeptide sequence, corresponding to 266–268 aa of human AT (LeuGlyGly) and BT (MetGlyAla), is conserved in the majority of GBGT1 gene-encoded FSs^10^. Mouse ABO gene-encoded *cis*-AB transferase also possesses the GlyGlyAla sequence^11^. Therefore, we examined its capacity to synthesize FORS1 by transfecting it into COS1(B3GALNT1) cells, which stably express human B3GALNT1 gene cDNA encoding β-1,3-*N*-acetylgalactosaminyltransferase 1 to increase globoside levels. We observed the appearance of FORS1, whereas human *cis*-AB transferase with LeuGlyAla tripeptide did not procure FS activity^12^. We also found FS activity in human AT (hAT) when substituting the LeuGlyGly tripeptide with GlyGlyAla. Those results demonstrated that the GlyGlyAla sequence, which resides in the catalytic center, is important for FS activity.

Previously, we found that deletion of exon 3 or 4 of hAT bestowed moderate FS activity^13^. We also observed that methionine to serine/threonine substitutions at aa position 69 (M69S/T) conferred weak FS activity^14^. Later, we also substituted the methionine residue at position 69 of the human AT by any one of the other 19 aa and found that many of the mutants, except for the original Met, Ile, Leu, and Phe substitutions, also conferred FS activity in COS-1 cells^15^. Furthermore, the co-introduction of exon 3 or 4 deletions or Met 69 mutations with the LeuGlyGly266-268GlyGlyAla substitution, corresponding to mFS, strengthened FS activity. The additive effect on FS activity of these mutations and their position in the stem region pointed to a catalytically independent mechanism of FS activity induction.

Golgi-residing glycosyltransferases, such as mFS and hAT, are type II transmembrane proteins with similar overall structures composed of a cytoplasmic tail, a membrane-spanning segment, a stem region, and a catalytic domain^16^. Various functions have been assigned to the stem region. First, it may serve as a flexible tether allowing the catalytic domain to glycosylate soluble and membrane-bound molecules in the Golgi apparatus lumen^16^. Second, it contributes to the Golgi localization of the enzyme together with cytoplasmic or transmembrane (TM) domains, as demonstrated for the α-helical stem region of human α-1,6-mannosylglycoprotein 6-β-*N*-acetylglucosaminyltransferase^17^, for the stem region of rat β-galactoside α-2,6-sialyltransferase^18, 19^ or bovine β-1,4-galactosyltransferase 1^20^. Third, it is the area where cleavage occurs for the production of soluble glycosyltransferases found in the circulation and other body fluids. Some examples are β-galactoside α2,6-sialyltransferase^21^, glycoprotein α-1,3-galactosyltransferase^22^, or the ABO blood group AT^23^.

In the present study, we studied possible mechanisms for the appearance of heterologous FS activity by mutations in the hAT stem region. Heterologous synthesis of both FORS1 and A/B antigens has been described in cancer^24–27^. Therefore, understanding new mechanisms for the somatic emergence of improper catalytical activities is crucial to exploit these antigens for future therapeutic strategies^28^.

## Results

### Forssman synthase activity acquisition by hAT stem mutants is not related to changes in their subcellular localization

**Figure 2:**
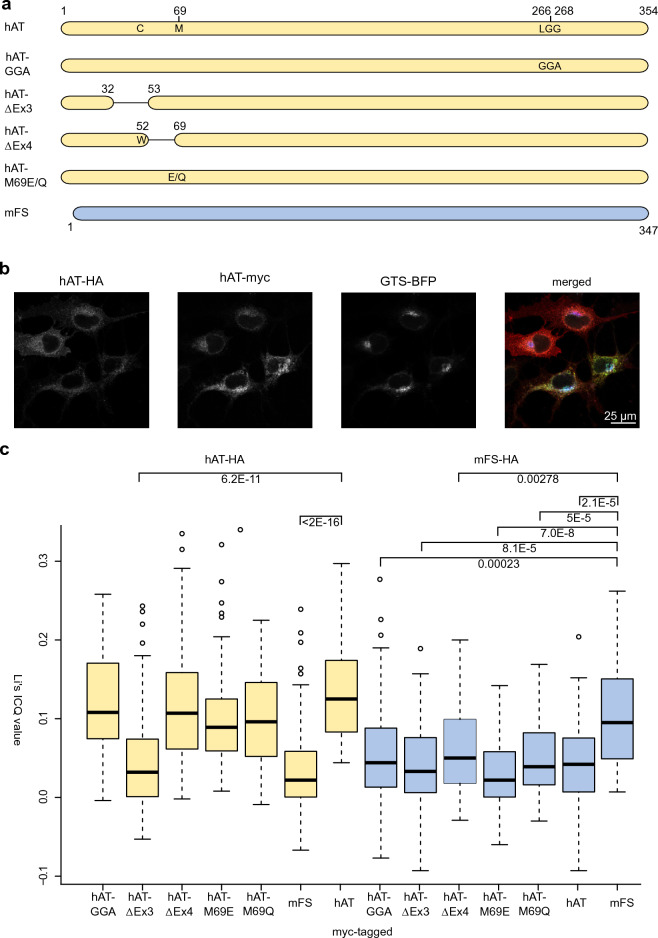
Colocalization of hAT mutants and hAT and mFS. Panel (**a**) shows a schematic representation of the proteins analyzed in the subcellular localization experiment. Amino acid positions are numbered, and original residues and mutations introduced are shown in a one-letter code. Panel (**b**) shows an example of the confocal images analyzed, the signals from HA and myc-tagged hAT, GTS-BFP, and the merged picture. Panel (**c**) consists of a boxplot of Li’s ICQ value for HA/myc-tagged protein pairs indicating their degree of colocalization within the Golgi apparatus. In yellow is shown the degree of colocalization of myc-tagged proteins with hAT-HA, and in blue, their colocalization with mFS-HA. Statistical significance between samples and controls is plotted with brackets, and p-values are included. For a complete analysis, see Supplementary Table S7.

We analyzed the effect of stem region mutations bestowing FS activity, exon 3 or 4 deletions and M69 mutations, and 266GlyGlyAla268 catalytic mutant in hAT intra-Golgi localization. For that purpose, we constructed plasmids coding for the original enzymes and mutants tagged with two different tags, myc, and hemagglutinin (HA), fused to their C-termini (Fig. 2a). Then, we transfected the myc-tagged mutants together with hAT-HA or mFS-HA and a blue fluorescence Golgi marker in COS-1 (B3GALNT1) cells and immunostained the cells with antibodies against these tags. Confocal images were taken. Figure 2 panel b shows an example of the optical sections obtained, in this case for hAT-HA and hAT-myc, together with GTS-BFP and the merged image.

To calculate the degree of colocalization within the Golgi, we used FiJi image analysis software, first creating regions of interest (ROI) corresponding to the GTS-BFP signal and analyzing the superimposition of myc and HA signals in those areas with the Coloc2 package. Then, the Li’s intensity correlation quotient (ICQ) (-0.5 segregation, 0 random colocalization, and 0.5 total colocalization) was plotted for each pair. The results are shown in Fig. 2c. All hAT mutants (hAT-GGA, hAT-ΔEx4, hAT-M69E, hAT-M69Q) colocalized with hAT in the same degree as wild-type hAT did with itself, except hAT-ΔEx3. Moreover, mFS did not colocalize with hAT. Reversely, all hAT mutants did not colocalize with mFS, including hAT-ΔEx3. Only mFS colocalized with itself.

The ΔEx3 mutation eliminates aa 33 to 52. Sequence analysis of hAT indicated the TM domain spans aa 20 to 37 (Phobius^29, 30^, DeepTMHMM^31^). The deletion eliminated the last five aa (G33, Y34, G35, and V36) of hAT TM α-helix, shortening the TM domain, which may explain this mislocalization.

### Analysis of previously identified FS-positive hAT mutants using in silico models reveal structural changes in the stem region

#### Exon deletion mutants

**Figure 3:**
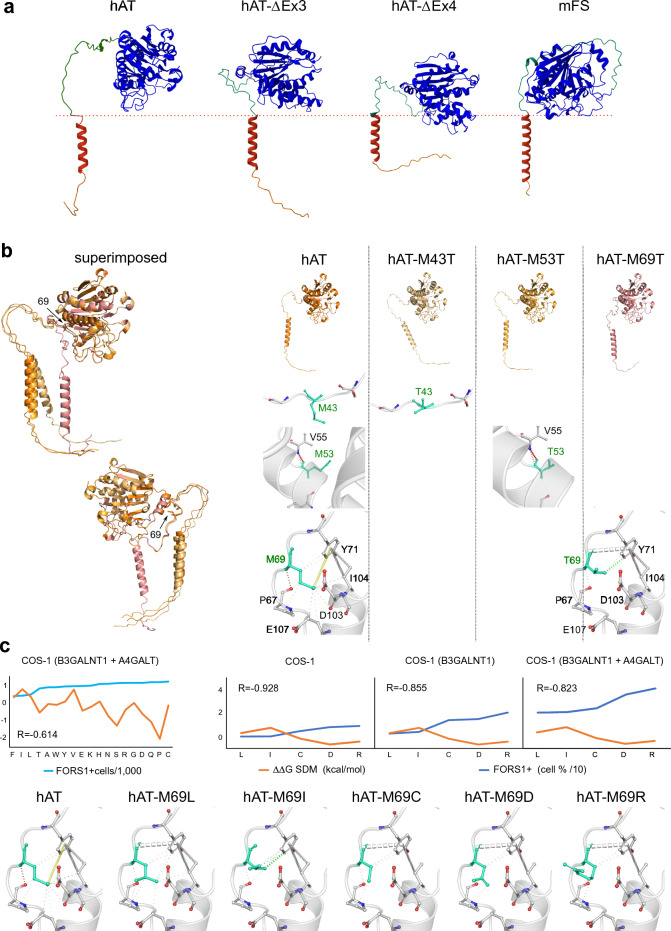
Structural analysis of hAT mutants with FS activity. Panel (**a**) shows the structural models for hAT and mFS obtained from the AlphaFold database, and for hAT-ΔEx3, hAT-ΔEx4 created using the ColabFold server. The catalytic domain, in blue, faces the right side, and the TM domain, in red, is oriented vertically. The green region between them corresponds to the stem region. Rendering was obtained using Mol* viewer. The red dotted line is a reference for the position of the internal luminal Golgi membrane layer. Panel (**b**) shows, on the left, the superimposed and aligned structural models of hAT and hAT mutants M43T, M53T, and M69T created with ColabFold. The arrows indicate the 69 position. The same structures are shown separately on the top right. Below are depictions of the interatomic interactions of aa occupying the 43, 53, and 69 positions. On the left column, Met corresponds to hAT, and in the following columns Thr for each mutant. Analyzed aa are shown in light green, and their interactions with surrounding aa were obtained using the DynaMut tool. The first graph in panel (**c**) shows, in light blue, the number of FORS1-positive cells detected by immunocytochemistry (divided by 1000 for visual clarity) of COS1 (B3GALNT1 + A4GALT) cells transfected with all M69X mutants and ranked from low to high positivity. In orange, the difference in free energy ΔΔG SDM (Site Directed Mutator, in kcal/mol) for each mutant is shown. R corresponds to the Pearson correlation coefficient between activity and ΔΔG SDM. The other graphs show, in blue, the percentage of FORS1 + positive cells (divided by 10) and, in orange, the ΔΔG SDM and the Pearson correlation coefficient between them. COS-1, COS-1 (B4GALNT1), and COS-1 (B3GALNT1 + A4GALT) cells were transfected with selected M69X mutants showing increasing FS activity measured by fluorescent cytometry. Interatomic interactions of aa at 69 position are shown for wild-type Met, and for cytometry-selected mutants and were calculated and rendered by the DynaMut server.

As no crystal structure has been solved for FS and for most of the non-globular domains of hAT (most structures at PDB, 1LZI^32^ for example, start at V64), we turned to AlphaFold structural database^33^ and compared the predicted structures of hAT and mFS. Additionally, we created models for hAT-ΔEx3 and hAT-ΔEx4 mutants using the ColabFold protein structure prediction software^34–36^. The proteins are shown in Fig. 3a.

AlphaFold2 and ColabFold predictions provide a confidence score per residue (pLDDT) indicating the prediction reliability^34, 37^. Scores below 50 correspond to unstructured regions and therefore the predicted structure in those areas is uninformative. The pLDDT for each model is illustrated in Supplementary Fig. S1.

The first model, hAT, shows the catalytic domain in blue (R81-P354). The catalytic domain start was assigned by multiple alignments of enzymes belonging to the glycosyltransferase family GT6 which coincided with the beginning of the sequence encoded by exon 6 (R81 in hAT)^38^. The TM domain is shown in red (L17-G35) and is perpendicularly oriented. We chose these TM limits by considering the structural prediction of hAT (L17-F32) and the sequence predictions abovementioned. The green region between them corresponds to the stem region, which we defined as V36 to C80 and displays a tether-like conformation, especially the V36-L61 segment which the prediction indicates is unstructured. Finally, in orange is the cytoplasmic N-terminal tail (M1-A16).

Next is hAT-ΔEx3. As discussed before, part of its TM domain was deleted. The structural model assigned the TM α-helix to aa L17 to V35 (corresponding to V55 in the full-length protein). Thus, 3 aa corresponding to the stem region of hAT were predicted to be in the TM domain. The stem region is 20 aa shorter and spans from R36 to C60 (corresponding to hAT R56 to C80). The pLTTD scores for A34 to H40 were below 50 and correspond to an unstructured area.

The structural model for hAT-ΔEx4 showed a shift in TM α-helix prediction (R18-V36). Therefore, we defined the TM as L17-G35 to maintain consistency with hAT. The stem region spans from V36 to R65 (corresponding to R56 to C80 with a 17 aa deletion from M53 to R68 of hAT) and contains an aa change, C52W, due to the altered exon boundaries. The region L37 to R49 is unstructured.

The last model is mFS. The predicted TM domain is larger than in hAT and includes most of the N-terminus (R5-L32). The catalytic domain was assigned by structural similarity to hAT (see Supplementary Fig. S2) and corresponded to P76 to T347. The stem region expands from V33 to G75. Only aa W30 (in TM domain) to Y38 (stem) were predicted to be unstructured.

The main difference between mFS and hAT was that mFS stem region surrounded the catalytic domain, while in hAT, the stem region was not as tightly associated with the catalytic domain, especially the first segment, from V33 to R63 (which is predicted to be unstructured). Moreover, the mFS stem region showed a short α-helix (P43-F46), which was close to two catalytic domain α-helixes (W176-E198 and V269-I283), that was absent in hAT.

All structural models for GBGT1-encoded proteins also displayed this compact stem configuration (see Supplementary Fig. S4). In contrast, the stem regions corresponding to orthologous ABO models acquired a tether conformation. There are some exceptions in which the stem region has been shortened, such as in mouse AB transferase or the rat B transferase, encoded by the Abo2 gene (see Supplementary Fig. S5).

In summary, hAT has a longer, more motile stem region with a large unstructured stretch (28 aa, Y34-L61). Second, the deletion mutants presented a shortened stem region (20 aa less for hAT-ΔEx3 and 17 aa for hAT-ΔEx4) and show smaller unstructured segments (7 aa for hAT-ΔEx3 and 13 aa for hAT-ΔEx4) restricting their deformability and bringing the catalytic domain towards the membrane. And third, mFS catalytic domain is located next to the TM domain, and presents only a very small unstructured segment in the stem-TM interphase, having the major part of the stem region enveloping the catalytic domain and interacting with it.

#### Methionine to threonine stem mutants

We obtained the structural models for hAT with Met to Thr substitutions in the stem region: M43T, M53T, and M69T. Only the last mutant possessed FS activity^14^. A superimposed image of the models obtained and individual ones are shown in Fig. 3b.

The M69T mutant stem region (shown in pink in Fig. 3b) does not align structurally with the other mutants or the wild-type protein. Using the DynaMut tool^39^, we found that many interatomic interactions in the wild-type structure between Met 69 and surrounding aa were lost. In wild-type, Met 69 sulfur atom interacts with the hydroxyphenyl ring of Tyr 71 (in yellow). This ring also showed hydrophobic interactions with two -CH- groups (C4, C5) of the Ile 104 side chain. Hydrophobic interactions between the methionine side chain and the side chains of Pro 67, Tyr 71, Asp 103, Ile 104, and Glu 107 were also found (see Fig. 3b). When Thr was introduced in this position, all these interactions were lost except the interactions with I104 and two new ones were formed: an amide-ring interaction between Thr 69 and Tyr 71 and a van der Waals-hydrophobic interaction between the Cγ of Thr 69 side-chain (in white) and the Cε1 of Tyr 71 ring (in green). No effects were found for the introduction of Thr in 43 and 53 positions.

#### M69X mutants

We calculated the stability difference between the wild-type and mutant proteins with every aa in position 69 using Site Directed Mutator (SDM) prediction^40^ and calculated the values of differential free energy. Activity measurements were obtained previously and showed that non-hydrophobic aa bestowed FS activity when transfected in COS-1 cells^15^. The first graph of Fig. 3c shows, in light blue, the number of FORS1 positive cells (divided by 1000 for graphic clarity) of COS1 (B3GALNT1 + A4GALT) cells transfected with all the mutants detected by immunocytochemistry and ranked from low to high FORS1 positivity, and in orange the corresponding difference in free energy (ΔΔG SDM, in kcal/mol). Positive values are a measure of protein structure stabilization induced by the mutation, while negative values correspond to destabilization. The Pearson correlation coefficient between activity and ΔΔG SDM showed a moderate negative correlation. In this experiment, cells were stained three days after transfection, so there was an accumulation of FORS1. The other graphs show the results of cytometry assays at 16 h post-transfection, which minimize this accumulation but only for selected mutants: M69L, I (lower or no FS activity), C (intermediate), D and R (higher). Again, in blue is shown the percentage of positive cells (divided by 10), and in orange, ΔΔG SDM. The analysis indicated that increased destabilization correlated with increased activity as Pearson correlation coefficients were -0,928 for COS-1 transfected cells, -0.855 for COS-1 (B4GALNT1), and -0.823 for COS-1 (B3GALNT1 + A4GALT). Interestingly, as the precursor, globoside, becomes more available by the overexpression of β3-*N*-acetylgalactosyl transferase 1 and α-4-galactosyltransferase (see Fig. 1), the correlation decreases slightly, which confirms the positive effect of higher precursor concentration on enzymatic activity.

We also predicted the mutants’ interatomic interaction changes using the DynaMut server (see Fig. 3c, bottom row). When Leu was introduced in the 69 position, a new interaction between the amide of the Leu 69 and the Tyr 71 ring was formed. However, others disappeared: the sulfur-ring interaction and the hydrophobic interactions with Asp 103, Glu 107, and partially with Ile 104.

For M69I, only the hydrophobic interactions of Y71 with I104 were conserved, while all others disappeared. One new hydrophobic van der Waals interaction was formed between the isoleucine side chain and the Y71 ring.

For M69C, M69D, and M69R, the amide-pi interaction between the aa at 69 and Y71 was saved as in the leucine mutant, but one of two hydrophobic between the Y71 ring and I104 side chain were lost. Therefore, increasing loss of interatomic interactions correlated with increased FS activity.

#### Normal mode analysis

Normal mode analysis (NMA) analyzes the overall functional motions of proteins or other macromolecules^41^. We used the AlphaFold structures for hAT and mFS and the tridimensional models obtained with ColabFold and performed NMA using the online tool iMODS^42, 43^.

**Figure 4:**
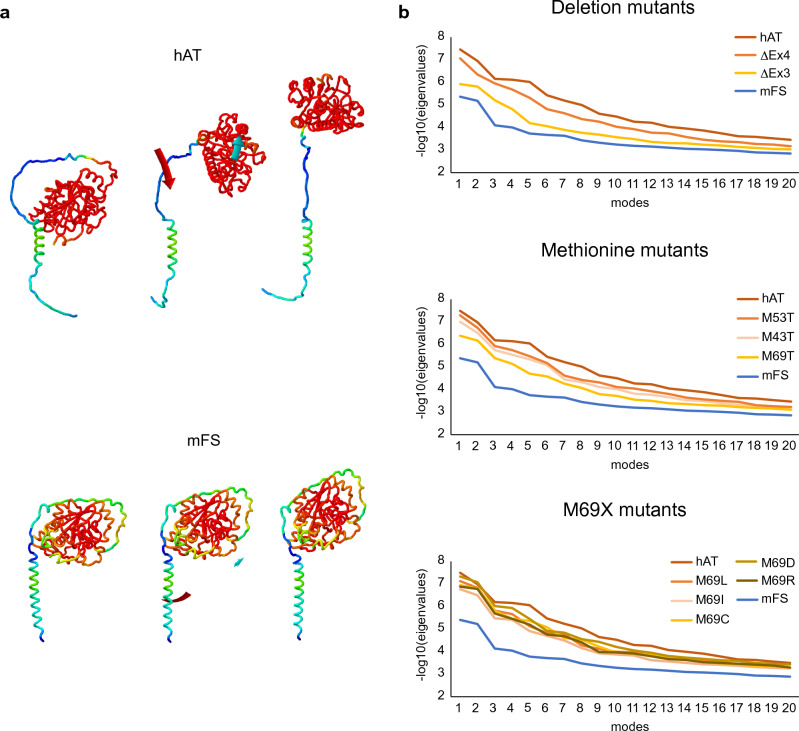
Normal mode analysis of mFS, hAT, and hAT mutants. AlphaFold structural models of hAT and mFS and models obtained by ColabFold for the hAT mutants were subjected to NMA using the iMODS tool. The depiction of the first vibrational mode for hAT and mFS is illustrated in panel (**a**). The two extreme conformations and an intermediate showing affine-arrows for the domains movements are shown. The spectrum coloring indicates the pLDDT score per residue (blue for low and red for high score) with a range of 28.59–98.93 for hAT and 39.16–98.81 for mFS. In panel (**b**), the eigenvalues for the first 20 modes were obtained for all protein models, and their negative common logarithm were calculated and plotted. The wild-type enzymes are shown in dark orange for hAT and blue for mFS, and the mutants are colored as found in the graph legends.

In Fig. 4 panel a, we show an illustration of the major normal vibration mode for hAT and mFS. It is evident that hAT has a much higher range of motion than mFS.

In order to show the range of motions for each mode, representing one given protein trajectory, the NMA results were summarized as eigenvalues. Negative eigenvalues correspond to higher deformability. In Fig. 4 panel b, we have plotted the negative common logarithm of the eigenvalues for hAT, its mutants, and mFS. We have used a logarithmic scale because of the very high numerical differences of eigenvalues between the modes. Accordingly, in this representation, the higher the value, the higher the protein mobility. As stated before, mFS was much less mobile, especially in the lower modes, while hAT displayed higher values, indicating a higher degree of protein mobility.

Deletion mutants, hAT-ΔEx3 and hAT-ΔEx4, displayed reduced mobility with respect to hAT in all modes. This was especially true for hAT-ΔEx3.

For hAT-M69T, it was evident that it had lower values than hAT, hAT-M43T, and hAT-M53T, especially for the first modes (1 to 4). However, when the mode increased, those differences were reduced.

Finally, all the values for the single aa substituted hAT (M69L, M69I, M69C, M69D, and M69R) were similar and lower than the wild-type enzyme.

### Selection of new stem region hAT mutants

**Figure 5:**
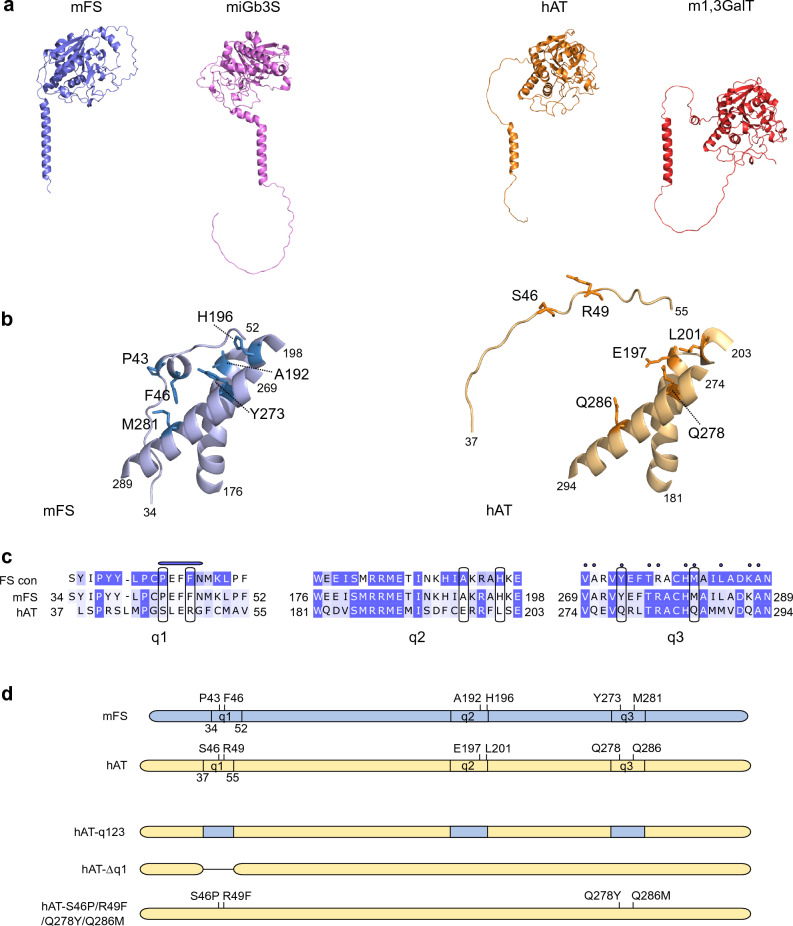
Tridimensional structure models of GT6 glycosyltransferase family members and selection of new mutations to induce FS activity. Panel (**a**) show the structural models for mFS, in blue, and mouse isoglobotriaosyl ceramide synthase (miGb3S), in violet, on the left; and hAT, in orange, and mouse glycoprotein α-1,3-galactosyltransferase (m1,3-GalT), in red, on the right. Panel (**b**) is a close-up of the central part of the stem region and interacting α-helixes in mFS, in blue, and the corresponding structures of hAT, in dark yellow. Specific mutations are annotated, and their side chains are displayed on the structure with a darker shade. Panel (**c**) corresponds to the protein sequence alignments of mFS and hAT for the segments presented above. A darker blue shade shows higher conservation. On top of the alignment, there is the consensus alignment of FORS1 + positive species (mouse, guinea pig, horse, dog, chicken) following the same color scheme. Black boxes encircle the mutated residues. The blue bar in the q1 segment corresponds to the short α-helix predicted in the mFS model, and the blue dots above the q3 segment to those residues in the α-helix point towards the stem region in mFS and hAT structures. Panel (**d**) shows  mFS, hAT and three mutants exemplifying the nomenclature of the hAT mutants constructed.

We also investigated the stem region conformation of other members of the α1,3-glycosyltransferase family, which are all evolutionary related^10^. Four representative members of this family are depicted in Fig. 5 panel a. On one hand, FS and α-1,3-galactosyltransferase 2 (also known as isoglobotriaosylceramide synthase) presented a stem region closely associated and encircling the catalytic domain^44^. Both enzymes use glycolipids as acceptor molecules. In contrast, AT and glycoprotein α-1,3-galactosyltransferase^45^, present a stem region not in contact with the globular domain and are known to modify both glycolipids and proteins.

Next, we centered our attention on a short α-helix predicted to form in the mFS stem region (P43-F46) that was absent in hAT (Fig. 5b).

Figure 5 panel c shows the protein alignment corresponding to three different segments, which we named conventionally q1 (mFS 34–52, hAT 37–55) corresponding to a fragment of the stem region, q2 (mFS 176–198, hAT 181–203) and q3 (mFS 269–289, hAT 274–294) both belonging to the catalytic domain. The first row corresponds to the FS consensus obtained by aligning FORS1-positive (FORS1 +) species FS (see Supplementary Fig. S2). Below, the aligned sequences of mFS and hAT are shown. In the case of q1, due to the unstructured nature of hAT segment, a T-COFFEE alignment was performed. For q2 and q3, the alignment resulting from the structural alignment of mFS and hAT is shown (see Supplementary Fig. S3).

In the q1 region, the small additional α-helix in mFS is indicated by a blue bar. Of the aa involved, only P43 and F46 were conserved in FS sequences and differed in hAT. Hence, we selected those aa to be mutated to the corresponding mFS aa to induce FS activity (S46P and R49F). In q2, we selected the mutations E197A and L201H because those positions were conserved in FS but were not in hAT. For q3, due to the higher conservation between hAT and mFS, we focused on the aa with side chains pointing towards the stem region (blue dots) and selected the Q278Y and Q286M mutations as those positions were conserved in FS proteins, and less conserved in hAT.

Once the regions/aa of interest were chosen, we constructed various hAT mutants to test their FS activity. Three types of mutants were created; chimeric mutants, substituting one or multiple q regions of hAT by their corresponding ones from mFS; the q1 deletion mutant (hAT-Δq1); and missense mutants substituting selected aa with their corresponding aa from mFS introduced one by one or in different combinations. Figure 5 panel d shows a scheme exemplifying the nomenclature of the constructs used in the following sections. All the mutants tested are shown in Table 1.

**Table 1:** FORS1 positive cells results of the cytometric analysis of COS1 (B3GALNT1) cells transfected with plasmids encoding for mFS, hAT, and all the mutants constructed.

Transfected DNA	Mean ± SEM	*P* (t-test vs. hAT)
**mFS**	**20.3 ± 2.5**	**1.97392E−08**
hAT	0.0	1
**hAT-Δq1**	**9.6 ± 3.4**	**1.94865E−05**
**hAT-q1**	**0.1 ± 0**	**0.000763326**
hAT-q2	0.0 ± 0	1
hAT-q3	0.0 ± 0	1
hAT-q12	0.0 ± 0	1
hAT-q13	0.0 ± 0	1
hAT-q23	0.0 ± 0	1
hAT-q123	0.0 ± 0	1
hAT-q1/Q278Y	0.0 ± 0	1
hAT-q1/Q286M	0.0 ± 0	0.143158425
**hAT-S46P/R49F**	**0.1 ± 0.0**	**0.013830611**
**hAT-E197A/L201H**	**0.1 ± 0.0**	**0.000450629**
**hAT-Q278Y/Q286M**	**0.2 ± 0.0**	**0.000969097**
hAT-S46P/Q278Y	0.0 ± 0	0.098000915
**hAT-S46P/Q286M**	**0.2 ± 0**	**0.000132472**
hAT-R49F/Q278Y	0.0 ± 0	1
hAT-R49F/Q286M	0.0 ± 0	0.050682274
hAT-S46P/R49F/E197A/L201H	0.1 ± 0	0.098000915
**hAT-S46P/R49F/Q278Y/Q286M**	**0.3 ± 0.1**	**0.000375446**
**hAT-E197A/L201H/Q278Y/Q286M**	**0.3 ± 0**	**1.46402E−07**
**hAT-S46P/R49F/E197A/L201H/Q278Y/Q286M**	**0.3 ± 0.1**	**5.3343E−11**
**hAT-S46P**	**0.1 ± 0**	**0.018440806**
hAT-R49F	0.0	1
**hAT-Q278Y**	**0.1 ± 0**	**0.000510859**
**hAT-Q286M**	**0.1 ± 0**	**0.016523645**
pSG5	0.0 ± 0	0.306183723
2ry	0.0 ± 0	0.350179481
No staining	0.0 ± 0	1

FORS1 positive cell percentages from the cytometric analysis are summarized in this table. Rows corresponding to FORS1 positive constructs appear in bold, and the mean and standard error of the mean of at least 3 independent experiments per sample are shown. Also, the Student’s test (alpha-level 0.05, two-legged, equal variance) p-values comparing the data to the hAT control samples are shown.

### New hAT stem region mutants acquired FS activity without altering their AT capacity

**Figure 6:**
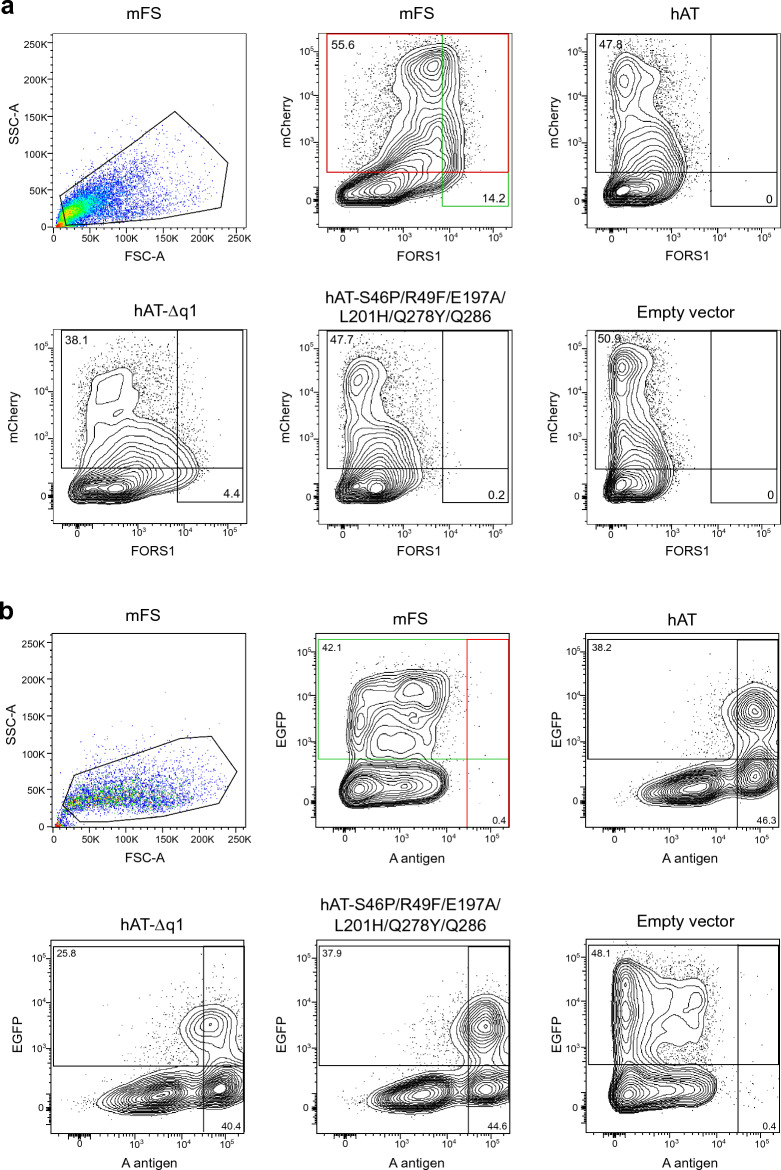
Forssman antigen detection by cytometric analysis of COS1 (B3GALNT1) cells and A antigen of HeLa (FUT2) cells transfected with plasmids encoding for mFS, hAT, selected hAT mutants, and the empty vector. In panel (**a**), cells were co-transfected with expression constructs and pmCherry-N1 plasmid. After 16 h, cells were detached and immunostained with anti-FORS1 and anti-rat Alexa Fluor 488 antibodies. The first graph shows the cell selection by forward (FSC) and side-scatter (SSC) amplitudes. Events are plotted in pseudocolor, with red representing the higher frequency and blue the lowest. The other graphs depict the density of cells plotted by their fluorescence values. The x-axis corresponds to 530 nm to detect FORS1, and the y-axis to mCherry protein fluorescence detected at 610 nm. Both these axes are on a logarithmic scale. The gates were set for each experiment using the positive and negative controls and maintained for all samples. Controls and selected samples from a representative experiment are shown. The percentages for FORS1 and mCherry-positive cells are given for each population. In panel (**b**), cells were co-transfected with the expression constructs and pEGFP-N1 plasmid. After 16 h, cells were detached and immunostained with anti-A antigen and anti-mouse Alexa Fluor 647 antibodies. The first graph shows the cell selection by forward (FSC) and side-scatter (SSC) amplitudes. Events are plotted in pseudocolor, with red representing the higher frequency and blue the lowest. The other graphs depict the density of cells plotted by their fluorescence values. The x-axis corresponds to A antigen detection at 660 nm and the y-axis to 530 nm to EGFP fluorescence. Both these axes are on a logarithmic scale. The gates were set for each experiment using the positive and negative controls and used for all samples. Controls and selected samples from a representative experiment are shown. The percentages for A antigen and EGFP-positive cells are given for each population.

FS activity was measured by the appearance of FORS1 on the surface of COS-1 (B3GALNT1) cells transfected with the various constructs and analyzed by flow cytometry. FORS1 + cells and mCherry (used as transfection control) positive cells were counted. Only samples with ≥ 20% mCherry-positive cells were considered. Assays were done 16 h after transfection. A representative example is depicted in Fig. 6a, showing the gating strategy. The results are summarized in Table 1.

First, we analyzed the results of the chimerical mutants: hAT-q1, q2, q3, q12, q23, q13, and q123. Mutant hAT-q1 was positive for FS activity (0.1% FORS1 + cells), and the deletion mutant hAT-Δq1 acquired a significant FS activity, 9.6% of FORS1 + cells, around a half of mFS positive control (20.3%).

Next, we tested missense mutations in various combinations. Three double mutants, hAT-S46P/R49F, S46P/Q286M, and E197A/L201H showed low but statistically significant FS activity (0.1% of FORS1 + cells), as did Q278Y/Q286M mutant (0.2% of FORS1 + cells).The introduction of more mutations as in hAT-S46P/R49F/Q278Y/Q286M, E197A/L201H/Q278Y/Q286M and S46P/R49F/E197A/L201H/Q278Y/Q286M increased FORS1-positive cells to 0.3% of, while hAT-S46P/R49F/E197A/L201H only achieved a 0.1% of positive cells.

Therefore, we tested hAT-S46P, R49F, Q278Y, Q286M single mutants. Of these, only S46P, Q278Y, and Q286M mutants showed statistically significant FS activity (0.1% of FORS1 + cells).

No activity was found for mutants combining the chimeric q1 mutation and single aa mutations.

Next, HeLa FUT2 cells^12^ were transfected with all FS-positive stem region hAT mutants, immunostained with anti-A and anti-mouse-IgM-Alexa Fluor 647 antibodies, and analyzed by cytometry. EGFP expression was used as transfection control. We followed a similar strategy to the one utilized to determine the FORS1 + cells, as seen in Fig. 6b.

The results indicated that all the FS activity-positive mutants had comparable AT activity and showed no statistical differences with hAT. Both negative controls, mGBGT1 and empty vector (pSG5) were A antigen negative, presenting only background levels. The results are summarized in Table 2.

**Table 2:** A antigen-positive cells results of the cytometric analysis of HeLa (FUT2) cells transfected with plasmids encoding for mFS, hAT, and all the mutants constructed.

Transfected DNA	Mean ± SEM	*P* (t-test vs. empty)	*P* (t-test vs. hAT)
mFS	0.5 ± 0.1	0.23291843	2.09901E−09
hAT	49.1 ± 3.4	2.02136E−09	1
hAT-Δq1	45.9 ± 4.7	4.66577E−08	0.539164234
hAT-q1	44.2 ± 13	0.000125203	0.674804735
hAT-S46P/R49F	44.5 ± 14	0.000196325	0.716057932
hAT-E197A/L201H	48.8 ± 6.1	2.17667E−07	0.95209349
hAT-Q278Y/Q286M	50.9 ± 4.0	5.69178E−09	0.697705142
hAT-S46P/Q286M	51.1 ± 4.9	2.65211E−08	0.703396561
hAT-S46P/R49F/Q278Y/Q286M	53.0 ± 6.7	2.31877E−07	0.567448575
hAT-E197A/L201H/Q278Y/Q286M	52.0 ± 7.6	6.80228E−07	0.691649536
hAT-S46P/R49F/E197A/L201H/Q278Y/Q286M	47.1 ± 3.8	7.34892E−09	0.648115459
hAT-S46P	57.9 ± 2.0	9.29996E−12	0.053869496
hAT-Q278Y	49.8 ± 3.8	4.51193E−09	0.881131483
hAT-Q286M	51.6 ± 1.0	7.15733E−14	0.447447772
Empty vector	0.3 ± 0.1	1	2.02136E−09
Secondary control	0.0	0.004451358	3.29552E−07
No staining	0.0	0.004451358	3.29552E−07

A antigen-positive cell percentages from the cytometric analysis are summarized in this table. The mean and standard error of the mean of at least 3 independent experiments per sample are shown. Also, the Student’s test (alpha-level 0.05, two-legged, equal variance) p-values comparing the data to the empty vector and hAT samples are shown.

## Conclusions

We first tested the possibility that stem region mutations ΔEx3, ΔEx4, M69E, and M69Q caused a change in subcellular localization of hAT within the Golgi apparatus, bringing it closer to where FS is located, which might correspond to a more favorable environment for the appearance of heterologous FS activity. The existence of specific locations for Golgi glycosyltransferases as means for specific, improved, and efficient biosynthesis of glycans has been proposed^46, 47^. Nonetheless, the results indicated that hAT and mFS subcellular localization within the Golgi is not playing a crucial role in inducing the appearance of FS activity in hAT-ΔEx3, hAT-ΔEx4, hAT-M69E, and hAT-M69Q mutants. Another interesting finding was the loss of colocalization of hAT-ΔEx3 with hAT, probably due to the partial deletion of the TM domain.

We then turned to hAT and mFS AlphaFold tridimensional models, as hAT crystallographic structures only partially covered the stem region^32^. In addition, no structures have been published for any FS. The most obvious difference between the structural models was the distinct association of the stem region with the catalytic domain in mFS and hAT. While in mFS, the stem was completely associated with the catalytic domain encircling it, the stem region in hAT was not associated and was predicted to be unstructured between the end of the TM domain and M69. This was also supported by normal mode analysis of the models, which indicated that the range of movement of hAT was much higher than for mFS.

These differences were maintained for paralogous FS and A/B transferases models studied. Also, when we turn to other members of the GT family, both FS and isoglobotriaosylcermide synthase (encoded by A3GALT2) showed the same compact conformation. These enzymes use glycolipids as acceptors^44^. Therefore, they require the catalytic domain to be close to the membrane and a reduced capacity for this domain to move away from it. In contrast, glycoprotein α-1,3-galactosyltransferase (encoded by GGTA1), responsible for synthesizing the α-1,3-Gal epitope, has a stem region with a considerable portion that is not associated with the catalytic domain. This enzyme acts on glycoproteins and glycolipids, similarly to A/B transferases^45^. We can conclude that for the GT6 family, those enzymes acting exclusively on glycolipids present a stem region encircling the catalytic domain. In contrast, a motile tether stem region allows the enzymes acting on glycolipids and glycoproteins to bring its catalytic domain to their various substrates, both on membrane-bound or soluble molecules.

Phylogenetic trees show that ABO and GBGT1 are closely related, while A3GALT2 and GGTA1 genes are more similar^10^. Therefore, the association/dissociation of the stem region with the catalytic domain happened twice, once in each evolutionary branch, highlighting the role of this configuration changes in the divergence of α-1,3 glycosyltransferases.

The tridimensional models of hAT-ΔEx3 and hAT-ΔEx4 deletion mutants and their NMA indicated that their stem region exhibited an intermediate state between hAT and mFS, with shorter and less deformable stem regions which correlated with their capacity to synthesize FORS1.

A shorter stem region is not exclusive to these engineered mutants. Some AT orthologues also have a reduced stem such as mouse AB transferase protein^11^ which also shows intrinsic FS activity^14^. However, because it also harbors the GlyGlyAla tripeptide in its catalytic domain, as does FS, we cannot discern to which degree the shorter stem contributes to its FS activity. Rat B-transferase is encoded by an independent gene (Abo2) instead of an allelic form, as in humans, and also has a shortened stem region^48^. Thus, the stem region length has been altered through rodent evolution, and we can speculate that their specificity towards glycoproteins and glycolipids has shifted towards glycolipids. Quantitative studies have to be performed to confirm this hypothesis.

When methionines located in the stem region were mutated to Thr, it was found that hAT-M69T could synthesize FORS1^14^. In this work, we tested how these mutations (M43T, M53T, and M69T) affected the predicted structure of hAT. The models obtained when these mutations were introduced were similar except for M69T, which showed a twisted stem configuration and a loss of interatomic interactions at the 69 position. We extended this discovery to all the other aa. We found that all substitutions induced FS activity except hydrophobic side-chain aa (M, L, I, F), which did not promote FS activity in COS-1^14, 15^. Similar to the M69T mutant case, the structural analysis showed that the introduction of non-hydrophobic aa also disrupted the interatomic interactions between the 69 position and surrounding aa in varying degrees.

Overall, we found a correlation between a global loss of interatomic interactions of aa 69 and an increase in FS activity of the mutant, which also correlated with a lower ΔΔG SDM. We conclude that mutations at this location affected the structural stability and deformability of the enzyme and promoted a favorable orientation of the catalytic domain towards the membrane-located globoside inducing the production of FORS1 antigen.

We also created other single aa mutants with FS activity. First, we substituted the q1 region (37–55) with the corresponding sequence of mFS and achieved low FS activity. The fact that chimeric mutants other than q1 did not induce FS activity may indicate a complex structural relationship between the stem region and the rest of the protein. When q1 was deleted, higher activity was detected in consonance with hAT-ΔEx3 and hAT-ΔEx4. Finally, three missense mutations, one in the stem region: S46P and two in the 274–294 α-helix: Q278Y and Q286M, whose side-chains are oriented towards the stem region, were also positive for FS activity, and their effects were additive. Although we could not produce a substantial increase, the results demonstrate that mutations affecting this area other than M69X can induce activity. We did not detect any effect in AT activity by introducing stem mutations, but subtle differences may exist, which could be made apparent in other experimental systems^49^.

The capacity of hAT to synthesize FORS1 is enhanced when deletion mutations or stem region single aa mutants are acquired. In the context of a high somatic mutation rate in cancer, it is not farfetched to hypothesize that such mutations could be responsible for the appearance of heterologous FORS1 antigen in tumor tissues as it is described in the literature^25, 27, 50^. Various missense somatic mutations in the hAT stem region (14 different ones found in 16 instances) are annotated in the COSMIC database^51, 52^.

In conclusion, the stem region of hAT is crucial for its acceptor specificity, and its alteration induces structural changes promoting heterologous FS activity, which may have biological implications in cancer and other pathophysiological conditions.

## Methods

### Protein alignments, structural predictions, and normal mode analysis

We downloaded the following structural models from the AlphaFold database (https://alphafold.ebi.ac.uk)^37^: AF-P16442-F1-model_v2 (hAT) and AF-Q8VI38-F1-model_v2 (mFS), AF-Q3V1N9-F1-model_v4 (mouse isoglobotriaosylceramide synthase) and AF-P23336-F1-model_v4 (mouse glycoprotein α1,3-galactosyltransferase).

We also used the AlphaFold predictions to create mutant models using the collaborative platform ColabFold^34–36^. We used the default settings and selected the highest-ranked model (highest probability).

The effects of point mutations were studied using the DynaMut prediction server (https://biosig.lab.uq.edu.au/dynamut/. ^39^SDM was used to determine the ΔΔG of M69X mutagenesis^40, 53^. Normal mode analysis was performed by iMODS (https://imods.iqfr.csic.es/) ^42, 43^.

All structural alignments and rendering were performed using PyMOL^54^ or Mol* viewer (https://molstar.org/viewer) ^55^. Protein sequence alignments were performed using T-COFFEE (https://tcoffee.crg.eu/apps/tcoffee/do:regular) ^56^ and visualized by JalView^57^.

### Molecular biology

Standard molecular techniques were used throughout. Plasmid pRK5-B4GALT1(1–81)-mTagBFP2 CDS was created by switching the fluorescent protein of pRK5-B4GALT1(1–81)-EGFP^58^ by mTagBFP2 from and pmTag-BFP2 (Addgene) plasmid using *Bam*HI/*Not*I restriction sites.

Tagged mFS, hAT, and hAT constructs were constructed by amplifying the coding regions, eliminating the stop codon, and adding the required tag by PCR amplification using Pfx Supermix (Thermo Scientific), custom oligonucleotides and, as templates, pSG5-mGBGT1, pSG5-ABO(A) and its mutants already available in the laboratory^12–14^ and subsequently subcloned in pSG5 vector (*Eco*RI/*Bam*HI).

New mutants were created by the round-the-horn PCR technique^59^ using HS II Phusion mix (Thermo), custom oligonucleotides, and pSG5-ABO(A) as the initial template. The list of primers used can be found in Supplementary Table S5. After PCR amplification using the mutation-containing primers, the amplified DNA was excised from agarose gel and purified using the PureLink Quick PCR purification kit. The purified linear plasmid was phosphorylated with T4 PNK phosphorylase (NEB) at 37 °C in T4 ligase buffer (Thermo Scientific) and ligated overnight at 16 °C with T4 ligase in the same reaction tube.

All obtained constructs were Sanger-sequenced to rule out the presence of spurious mutations.

### Cells and transfection

COS1(B3GALNT1) cells and HeLa(FUT2) cells were used as recipients of DNA transfection and were previously described^12, 58^.

For the subcellular localization experiments, HeLa (FUT2) cells were seeded onto glass #1.5 coverslips precoated with 100 μg/ml poly-D-lysine and transfected using JetOptimus reagent (Polyplus) following the manufacturer’s instructions. 0.175 μg of each myc and HA construct and 0.15 μg of pGTS-BFP were introduced per sample. After 16 h, cells were fixed with paraformaldehyde 4%.

COS1(B3GALNT1) cells or HeLa (FUT2) cells were seeded on 12-well plates for cytometry experiments. The day after reaching around 80–90% confluence, cells were transfected using JetOptimus transfection reagent following the manufacturer’s instructions. 0.9 μg of sample DNA and 0.1 μg of pmCherry-N1 or pEGFP-N1 were transfected per well. The cells were analyzed 16 h post-transfection.

### Immunocytochemistry

Cells grown on glass coverslips were permeabilized and blocked using phosphate-buffered saline (PBS) plus 3% bovine serum albumin and 0.01% saponin. Tags were detected by immunostaining with anti-myc epitope antibody (9E10, Merck Millipore) and AlexaFluor-488 anti-mouse IgG antibody (Thermo Scientific), and polyclonal anti-HA (HA.11, Biolegend) and anti-rabbit-Alexa 594 antibody (Thermo Scientific). The coverslips were mounted with Mowiol 4–88.

### Microscopy and colocalization analysis

Confocal microscopy was used to determine the subcellular localization of myc/HA-tagged constructs and the GTS-BFP construct. Only one optical plane image was obtained per field using an LSM710 AxioObserver Zeiss microscope and a Zeiss Plan-Apochromat 63x/1.40 oil DIC M27 objective. At least 50 pieces of Golgi apparatus were analyzed for each construct.

The obtained images were opened in FiJi software. After creating regions of interest covering the GTS-BFP signal using intensity thresholds, the level of colocalization between the HA and myc constructs was calculated using the Coloc2 plugin. The resulting Li’s ICQ parameter^60^ for each paired sample was obtained, and data was analyzed and plotted using R studio 1.2.5033.

### Immunocytometry

After 16 h, COS-1 (B3GALNT1) cells were detached and subjected to immunostaining. They were first incubated with anti-FORS1 monoclonal antibody (FOM-1, BioRad) followed by Alexa Fluor 488-conjugated goat anti-rat IgM (µ chain specific) secondary antibody (Thermo Scientific). Then the cells were subjected to cytometry analysis. BD LSRFortessa Analyzer (BD Biosciences) was used. After selecting single viable cells by forward and side scattering gating, the mRFP fluorescence was measured at 610 nm to identify co-transfected RFP expression and to determine the DNA transfection efficiency. FORS1 expression was detected at 530 nm.

To test for AT activity, 16 h after transfection, HeLa (FUT2) cells were detached and incubated with the mixture of murine anti-A monoclonal antibodies (Bioclone Anti-A, OrthoDiagnostic Systems) and then with Alexa Fluor 647-conjugated goat anti-mouse IgM (µ chain specific) secondary antibody (Thermo Scientific). Immunostained cells were analyzed by cytometry as described above for COS1(B3GALNT1) cells. In addition, A antigen-positive cell percentages were measured by detecting fluorescence at 660 nm, and GFP expression was monitored at 530 nm.

Cytometry data were analyzed and plotted using FACS Diva and FlowJo software.

### Statistical analysis

For the colocalization quantification, data was analyzed using R studio. ANOVA analysis was performed first (*P* = 0.00605), and afterward, pairwise comparisons using t-tests with pooled SD were performed. The Holm method was used for the p-value adjustments. The complete pairwise comparison can be found in Supplementary Table S7.

For cytometry results, percentages of positive cells obtained were averaged, and standard error of the mean and t-test values (alpha-level 0.05, two-legged, equal variance) were calculated using Excel (Microsoft).

### Supplementary Information

Supplementary Information.

## Data Availability

The datasets used and analyzed during the current study are available from the corresponding author upon reasonable request.
