# Supplementary material for: The stem region of group A transferase is crucial for its specificity, and its alteration promotes heterologous Forssman synthase activity
Source: Sci Rep. 2023 Aug 26;13:13996. doi: 10.1038/s41598-023-40900-4 (PMC10460411; doi:10.1038/s41598-023-40900-4)
Supplement: Supplementary file 1 — Supplementary Information. [file 41598_2023_40900_MOESM1_ESM.docx]

# Supplementary information

**
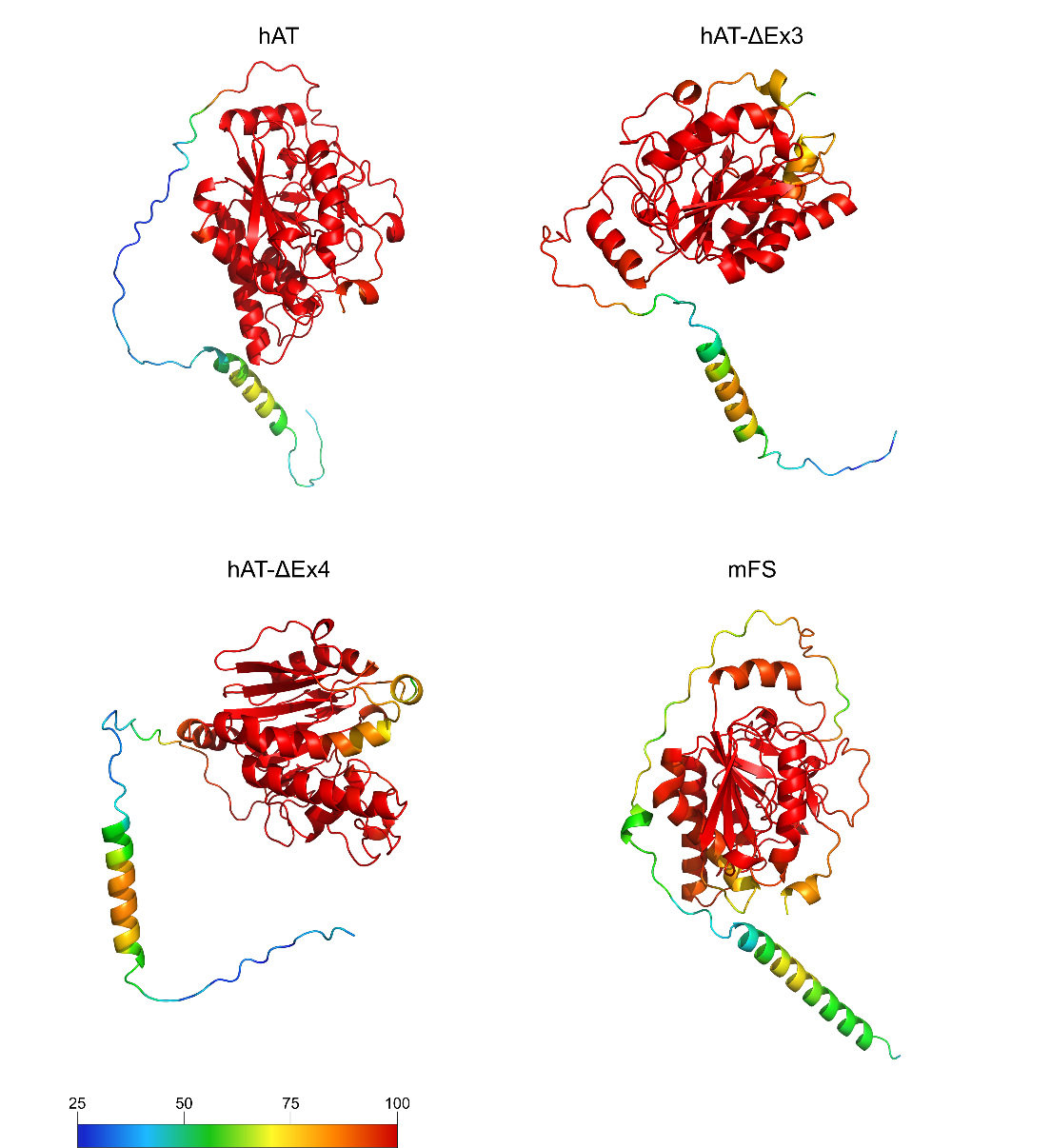
Figure S1. Prediction Confidence score of (pLDDT) of hAT, hAT-ΔEx3, hAT-ΔEx4 and mFS.**

The pLDDT score is produced by AlphaFold, and is a per-residue estimate of its confidence on a scale from 0 - 100. Here it is plotted by residue from 25 in dark blue to 100 in dark red. Regions with pLDDT > 90 (red) have high accuracy. Regions with pLDDT between 70 and 90 (yellow-orange) have a moderate accuracy and regions with pLDDT between 50 and 70 (greens) are low confidence although structured domains with many inter-residue contacts are likely to be more reliable than extended linkers or isolated long helices.

The 3D coordinates of regions with pLDDT < 50 (blue-cyan) should not be interpreted. These regions are unstructured in physiological conditions or only structured as part of a complex.

**Figure S2. T-COFFEE alignment of FS active proteins**


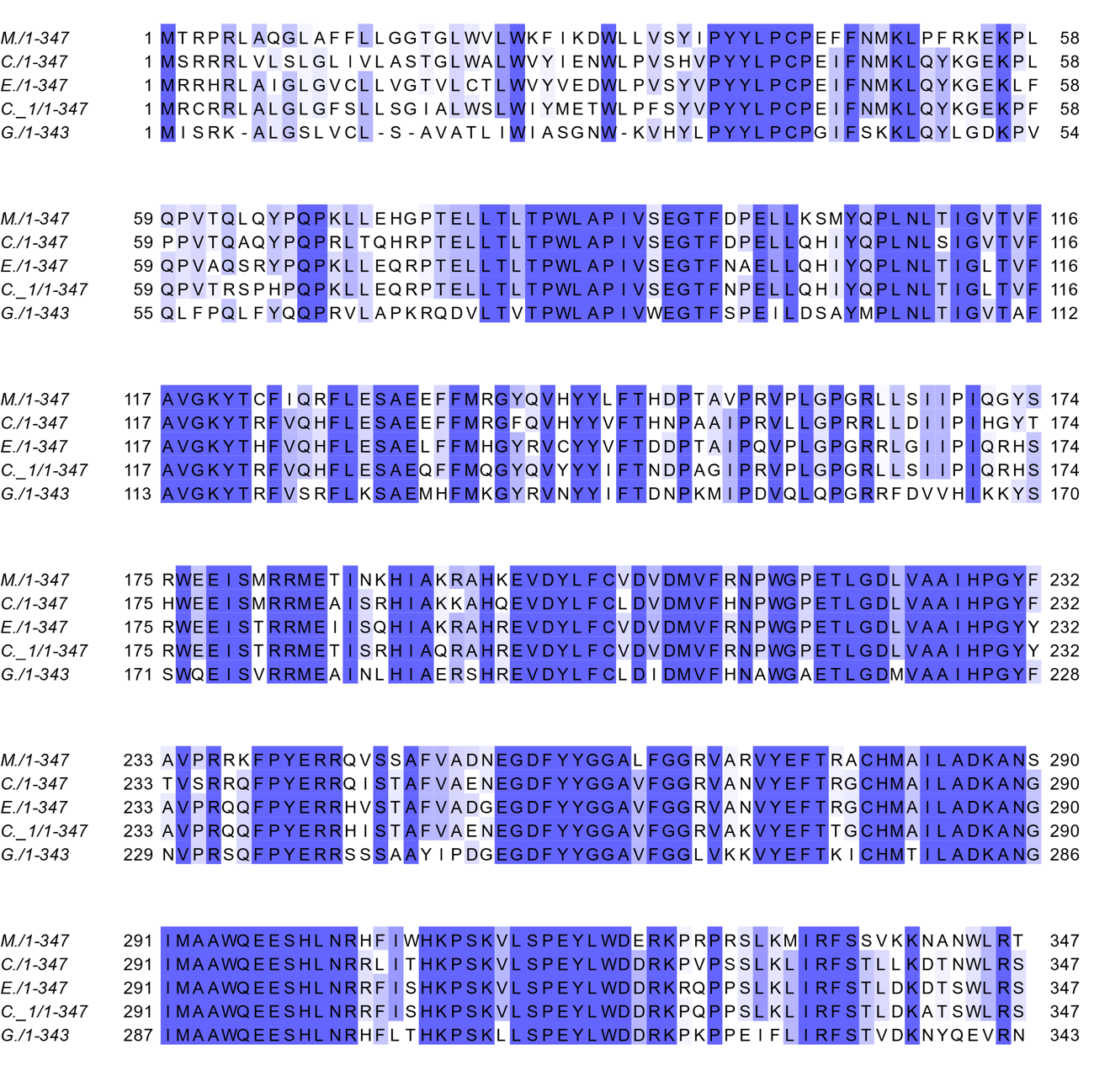


M. Mouse. Mus musculus. Uniprot Q8VI38

C. Guinea pig. Cavia porcellus. A0A286XG71

E. Horse. Equus caballus F6SY78

C._1 Dog. Canis lupus familiaris. Q95158

G. Chicken. Gallus gallus. Q5ZLK4

**Figure S3. Sequence alignment corresponding to the structural alignment of AlphaFold models**

After the structural alignment of mFS (AF-Q8VI38-F1-model_v2) and hAT (AF-P16442-F1-model_v2) models in PyMol, the resulting sequence alignment was visualized in JalView. Note that the N-termini were not aligned in the 3D models.


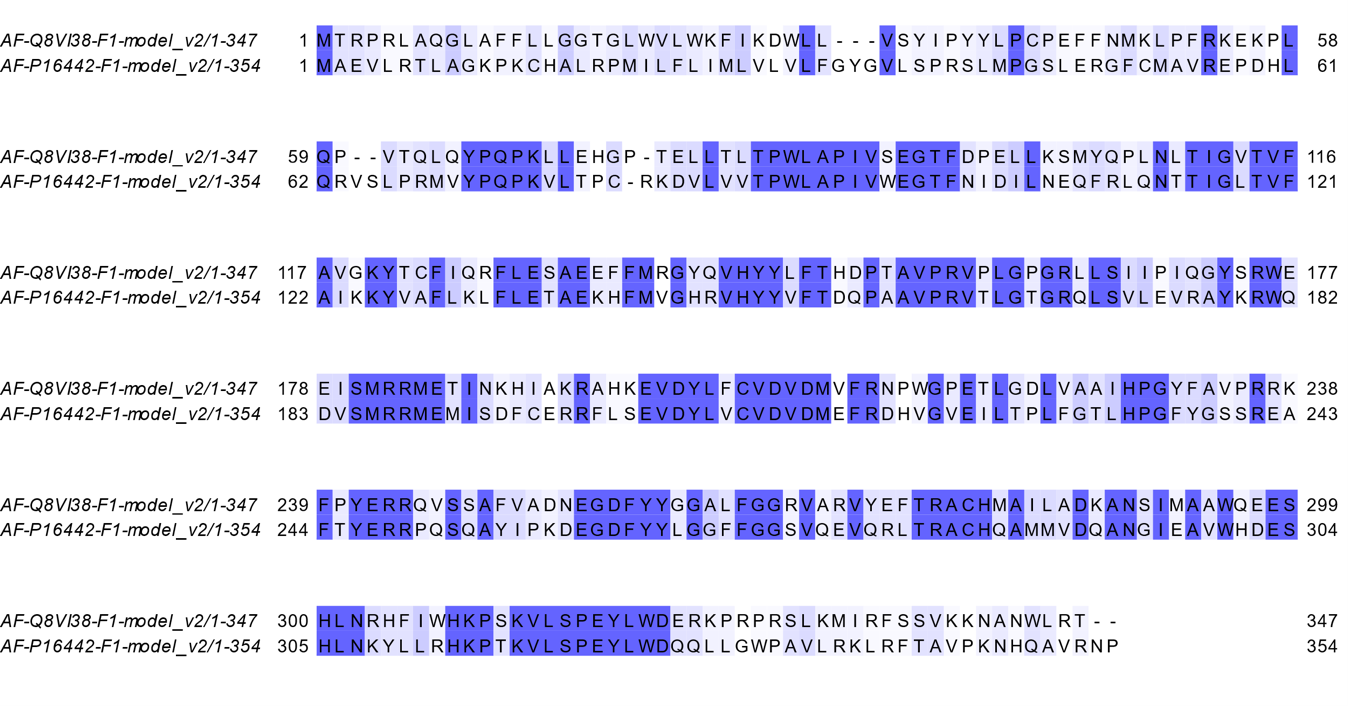


**Figure S4. Aligned Forssman synthases AlphaFold structural models.**


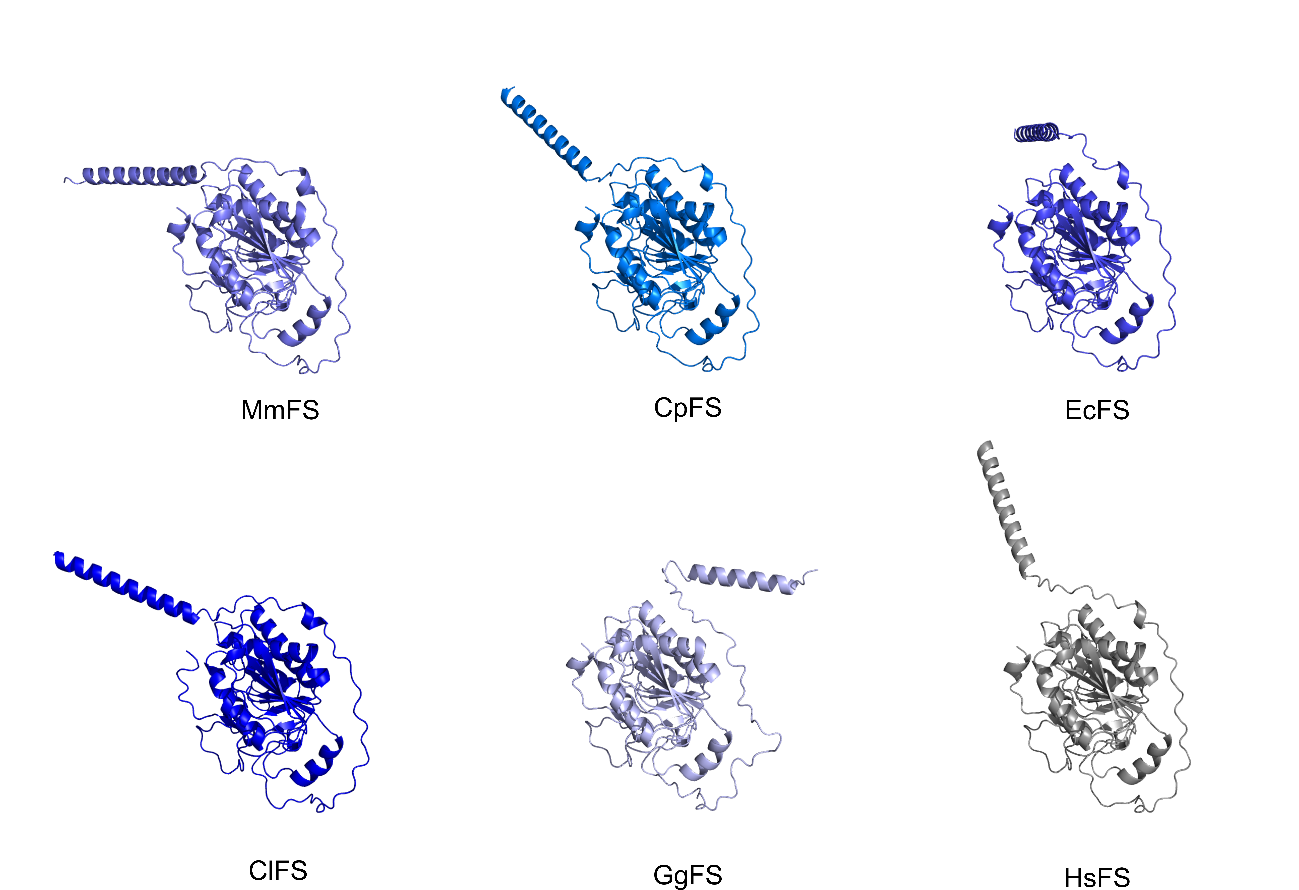
The AlphaFold models for mouse, guinea pig, horse, dog, chicken and human FS (AF-Q8VI38-F1-model_v2, AF-A0A286XG71-F1-model_v4, AF-F6SY78-F1-model_v4, AF-Q95158-model_v4, AF-Q5ZLK4-model_v4 and AF-Q8N5D6-F1-model_v2 respectively) were aligned using PyMol and show the stem region encircling the catalytic domain.

**Figure S5. Aligned A/B transferases AlphaFold structural models.**

The AlphaFold models for hAT, rat AT, mouse AB transferase and rat B transferase (AF-P16442-F1-model_v2, AF-Q9ET32-F1-model_v4, AF-P38649-F1-model_v4, and AF-P16442-F1-model_v2 respectively) were aligned using PyMol, on the left the models with full-length stem region and on the right with a shorter stem.


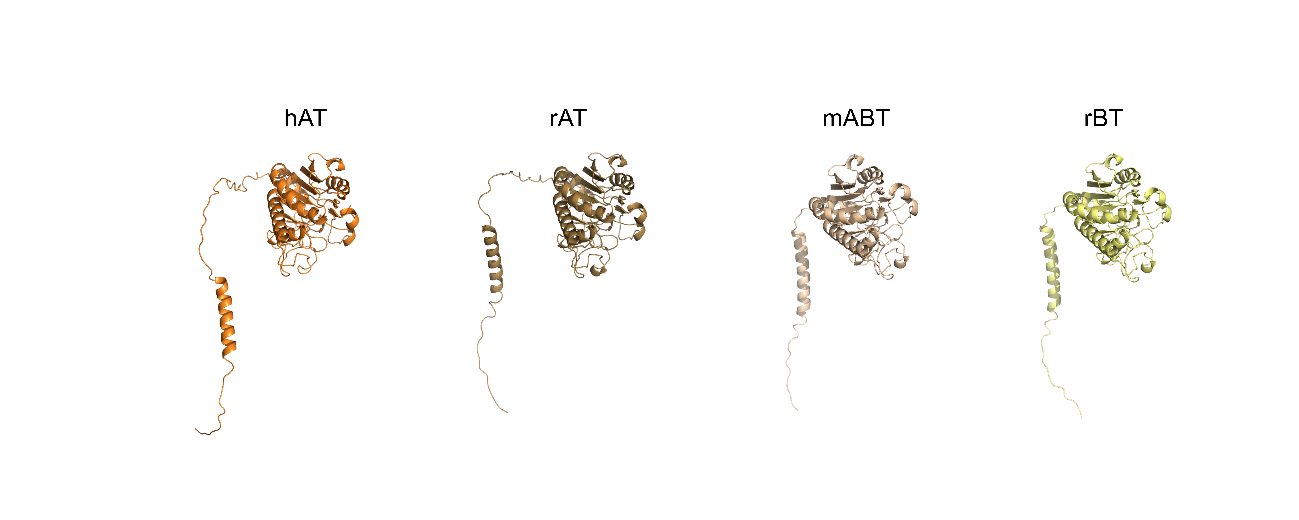


**Table S6. Oligonucleotides used for mutagenesis and cloning**

| Mutation | Oligonucleotides (forward above, reverse below) |
| --- | --- |
| S46P | CCCCTGGAACGGGGGTTCTGCAT  TCCTGGCATTAGACTTCTGGGGCT |
| R49F | TTCGGGTTCTGCATGGCTGTTAGGG  TTCCAGGCTTCCTGGCATTAGACTT |
| S46P/R49F | CCCCTGGAATTCGGGTTCTGCATGGCTGTTAGGGA  TCCTGGCATTAGACTTCTGGGGCT |
| E197A/L201H | CACGGCGCTTCCACAGCGAGGTGGATTACCTGGTGTG  CGCAGAAGTCACTGATCATCTCCATG |
| Q278Y | TACAGGCTCACGCGTGCCTGCC  CACCTCTTGCACCGACCCCC |
| Q286M | ATGGCCATGATGGTCGACCAGGC  GTGGCAGGCACGCGTGAGC |
| Q278Y/Q286M | TATCGGCTCACGCGTGCCTGCCACATGGCCATGATGGTCGACCAGGC  CACCTCTTGCACCGACCCCC |
| H301Q | GGACGAGAGCCACCTGAACAAGTACC  TGCCACACGGCCTCGATG |
| Δq1 | AGGGAACCTGACCATCTGCAGCG  GACCCCGTAACCAAACAAGACCAAGAC |
| q1 | CAGAGTTCTTCAACATGAAGCTTCCCTTCAGGGAACCTGACCATCTGCAGCG  GGCAGGGGAGGTAATAGGGTATGTAGGAGACCCCGTAACCAAACAAGACCAAGAC |
| q2 | TCAACAAACACATTGCCAAGAGAGCCCACAAAGAAGTGGATTACCTGGTGTGCGTGGAC  TGGTCTCCATTCGGCGCATGGAGATCTCCTCCCAGCGCTTGTAGGCGCGCAC |
| q3 | TGCCACATGGCCATTCTGGCAGACAAAGCTAACGGCATCGAGGCCGTGT  GGCCCGGGTAAACTCATACACTCTGGCCACCGACCCCCCGAAGAACC |

| Amplicon | Oligonucleotides (forward above, reverse below) |
| --- | --- |
| HA-tagged hAT  constructs | TAATACGACTCACTATAGGG  TAGGATCCTCAAGCGTAATCTGGAACATCGTATGGGTACGGGTTCCGGACCGCCTGGTGG |
| HA-tagged mFS | TAATACGACTCACTATAGGG  TTGGATCCTTAAGCGTAATCTGGAACATCGTATGGGTAGGTCCTCAGCCAGTTGGCATTC |
| myc-tagged hAT constructs | TAATACGACTCACTATAGGG  TTAGGATCCTCACAGATCCTCTTCTGAGATGAGTTTTTGTTCCGGGTTCCGGACCGCCTGGTGG |
| myc-tagged mFS | TAATACGACTCACTATAGGG  TTAGGATCCTCACAGATCCTCTTCTGAGATGAGTTTTTGTTCGGTCCTCAGCCAGTTGGCATTC |

**Table S7. Statistical analysis of colocalization**

ANOVA

Df Sum Sq Mean Sq F value Pr(>F)

Colocconfoigtp$sample 1 0.035 0.03519 7.572 0.00605 **

Residuals 912 4.239 0.00465

---

Signif. codes: 0 ‘***’ 0.001 ‘**’ 0.01 ‘*’ 0.05 ‘.’ 0.1 ‘ ’ 1

Pairwise comparisons using t tests with pooled SD

data: Colocconfoigtp$`Li's ICQ value` and Colocconfoigtp$sample

1 2 3 4 5 6 7 8 9 10 11 12 13

2 1.7e-09 - - - - - - - - - - - -

3 1.00000 1.5e-09 - - - - - - - - - - -

4 1.00000 3.5e-05 1.00000 - - - - - - - - - -

5 1.00000 4.3e-05 1.00000 1.00000 - - - - - - - - -

6 8.5e-16 1.00000 5.1e-16 3.1e-10 1.1e-09 - - - - - - - -

7 5.6e-09 1.00000 5.0e-09 0.00014 0.00017 0.94204 - - - - - - -

8 5.8e-09 1.00000 5.6e-09 5.7e-05 6.2e-05 1.00000 1.00000 - - - - - -

9 6.7e-07 1.00000 6.7e-07 0.00232 0.00220 1.00000 1.00000 1.00000 - - - - -

10 4.7e-13 1.00000 3.7e-13 3.0e-08 5.4e-08 1.00000 1.00000 1.00000 1.00000 - - - -

11 2.0e-09 1.00000 1.9e-09 3.3e-05 3.9e-05 1.00000 1.00000 1.00000 1.00000 1.00000 - - -

12 5.7e-10 1.00000 5.1e-10 1.2e-05 1.6e-05 1.00000 1.00000 1.00000 1.00000 1.00000 1.00000 - -

13 1.00000 6.2e-11 1.00000 0.16754 0.45557 < 2e-16 2.0e-10 2.5e-10 3.0e-08 1.4e-14 7.7e-11 2.0e-11 -

14 1.00000 5.5e-05 1.00000 1.00000 1.00000 1.4e-09 0.00023 8.1e-05 0.00278 7.0e-08 5.0e-05 2.1e-05 0.31637

P value adjustment method: holm

1- hAT-HA/hAT-GGA-myc

2-hAT-HA/hAT-delEx3-myc

3-hAT-HA/hAT-delEx4-myc

4-hAT-HA/hAT-M69E-myc

5-hAT-HA/hAT-M69Q-myc

6-hAT-HA/mFS-myc

7-mFS-HA/hAT-GGA-myc

8-mFS-HA/hAT-ΔEx3-myc

9-mFS-HA/hAT-ΔEx4-myc

10-mFS-HA/hAT-M69E-myc

11-mFS-HA/hAT-M69Q-myc

12-mFS-HA/hAT-myc

13-hAT-HA/hAT-myc

14-mFS-HA/mFS-myc
